# Sex differences in music performance anxiety: the role of self/other-scrutiny and self-efficacy

**DOI:** 10.3389/fpsyg.2026.1746532

**Published:** 2026-01-28

**Authors:** Hua Wu, Zhen Li, Jian Sun

**Affiliations:** 1 School of Music and Dance, Xihua University, Chengdu, China; 2 The University of Waikato, Hamilton, New Zealand; 3 Performance College of Wuhan Conservatory of Music, Wuhan, China

**Keywords:** Chinese choir members, music performance anxiety, other-scrutiny, self-efficacy, self-scrutiny, sex differences

## Abstract

**Introduction:**

Sex differences in music performance anxiety (MPA) remain a persistent concern in both research and practice, yet the mediation mechanisms are not fully understood. Thus, this study examined whether self-scrutiny, other-scrutiny, and self-efficacy mediate the relationship between sex and MPA among Chinese choir members.

**Methods:**

A cross-sectional online survey was conducted in June 2025 with 774 active participants (27.1% male; mean age = 46.02, SD = 18.19) recruited from four community-based choirs in China. Standardized measures assessed MPA, self-scrutiny, other-scrutiny, and self-efficacy.

**Results:**

Findings indicated that (1) females reported significantly higher MPA than males; (2) Self-scrutiny, other-scrutiny, and self-efficacy significantly mediated the sex-MPA association, with effect sizes of 36.0%, 16.3%, and 2.8%, respectively. (3) Serial mediation via self-scrutiny and self-efficacy was not supported, whereas the indirect pathway through other-scrutiny and self-efficacy was significant, though modest in size (1.0%).

**Conclusion:**

These findings highlight that evaluative cognitions, especially self-scrutiny, play a central role in explaining sex disparities in MPA, whereas self-efficacy exerts a limited contribution. In the Chinese choral context, where collective and public performance accentuates external evaluation, other-scrutiny may further erode confidence over time. The study underscores the need for interventions that target maladaptive self- and other-focused cognitions, while simultaneously fostering mastery experiences and supportive feedback to strengthen self-efficacy.

## 1 Introduction

Music performance anxiety (MPA) is a widely documented psychological phenomenon that afflicts both professional and amateur performers. It is characterized by heightened worry, physiological arousal, and impaired performance in evaluative musical contexts (Kenny, 2023). MPA not only diminishes performers’ artistic expression but also compromises their long-term well-being, motivation, and career development (Kinney et al., 2025). Choir singing, a highly social and evaluative form of performance, provides a unique context in which MPA emerges with particular intensity. Unlike solo performance, choir participation simultaneously emphasizes collective harmony and individual contribution, exposing members to dual evaluative pressures.

Sex disparity in mental health is an important research topic. In general, the prevalence of mental distress (e.g., anxiety) is higher among females than males (Lu et al., 2024a, 2024b; Shawon et al., 2024; Kayrouz et al., 2025). In the context of MPA, numerous Western studies also suggest that females report higher performance-related anxiety than males across diverse samples and cultural settings. Elevated MPA among female musicians has been observed in adolescent learners (Papageorgi, 2020), higher education music students in Portugal (Barros et al., 2024), university music students in Turkey (Cornett and Urhan, 2021), mixed samples of European musicians (Butković et al., 2021), and classical music students (Sokoli et al., 2022). However, this pattern is not entirely consistent. For example, a study of younger musicians aged 7–17 years found no significant sex differences in MPA (Dempsey and Comeau, 2019), suggesting that sex effects may vary across developmental stages, performance contexts, or methodological approaches. Despite the predominance of Western evidence, research specifically examining sex differences in MPA among Chinese musicians remains scarce. Therefore, it is important to test whether commonly reported sex differences in MPA generalize to Chinese choir singers, an understudied group.

Although sex differences in MPA are well documented, the underlying mechanisms remain unclear. Evaluative scrutiny may be a plausible cognitive mediator of such differences. Scrutiny in MPA involves heightened awareness of both internal standards and external judgments (Dobos et al., 2019). Crucially, we distinguish two conceptually separable forms of scrutiny from the outset. Self-scrutiny refers to an internal evaluative focus (e.g., monitoring one’s adequacy against personal standards, heightened self-criticism, and post-performance rumination), whereas other-scrutiny refers to a perceived external evaluative focus (e.g., concern about being negatively judged by audiences, peers, or the conductor) (Kenny, 2023). This distinction is especially relevant in choir settings, where performers may simultaneously evaluate themselves against internal standards while also feeling observed and judged within a socially dense group context (Du et al., 2025). Because these evaluative foci are triggered by the immediate performance situation, they offer a more context-sensitive account of MPA than broader, trait-like constructs that may not differentiate the specific sources of evaluation pressure in choral performance (Guyon et al., 2022). In addition, separating internal self-evaluation from perceived external judgement allows a more precise test of which aspect of evaluative concern is more consequential for sex differences in MPA, rather than treating “evaluation” as a single undifferentiated factor.

It is possible that females experience higher levels of self- and other-scrutiny than males, although direct empirical evidence remains limited. Gender role theory (Chaplin, 2015) offers a plausible explanation for this tendency: females are often socialized to be emotionally expressive, interpersonally responsive, and attentive to external approval. These gendered expectations can foster greater self-monitoring and sensitivity to evaluation, which in turn may intensify both self- and other-scrutiny in performance contexts (Kring and Gordon, 1998; Nicholl and Abbott, 2024). Such heightened scrutiny is likely to exacerbate vulnerability to MPA. Thus, it is expected that self/other-scrutiny may mediate the association between sex and MPA.

Self-efficacy is another plausible mediator between sex and MPA. According to gender role theory (Eagly and Wood, 2016), gendered socialization tends to reinforce agency, confidence, and achievement-oriented behaviors more strongly in males, whereas females are more often encouraged to prioritize relational responsiveness and emotional attunement (Yu and Deng, 2022). Such differences in social expectations may translate into modest sex differences in task-related self-efficacy (Moraga-Pumarino et al., 2025). Consistent with this account, empirical meta-analytic evidence supports modest but reliable gender differences in self-efficacy, with males often reporting somewhat higher confidence in domains traditionally viewed as male-typed (Yu and Deng, 2022). However, evidence for sex differences in self-efficacy is mixed, with some work in younger musicians reporting no significant differences (Dempsey and Comeau, 2019). Although prior work has not directly examined whether sex differences in MPA are explained through differences in self-efficacy, existing evidence offers indirect support for this possibility. For instance, studies in high-stakes settings show that lower self-efficacy is associated with higher performance anxiety (Malespina et al., 2024). Therefore, self-efficacy is examined as a theoretically plausible mechanism reflecting performers’ coping capacity under evaluation, despite limited direct evidence in the MPA literature.

Self- and other-scrutiny is also conceptually related to self-efficacy, suggesting the possibility of a sequential mediation. Excessive self-scrutiny and concerns about external evaluations may undermine confidence in one’s ability to manage performance demands (Herman and Clark, 2023; Kenny, 2023). According to the cognitive interference model, evaluative self-focus diverts attentional resources away from task-relevant processes, thereby reducing perceptions of competence and self-control (Wine, 1971; Eysenck et al., 2007). As self-efficacy reflects beliefs in one’s ability to succeed, evaluative scrutiny may weaken self-efficacy by amplifying doubts and increasing anticipatory worries (Jia and Yue, 2023). Empirical evidence shows that greater fear of negative evaluation correlates with lower self-efficacy in academic and performance contexts (Jia and Yue, 2023). In turn, low self-efficacy is consistently associated with higher levels of MPA (Zhang and Jenatabadi, 2024; Ou and Qin, 2025). Hence, it is plausible that sex differences in MPA are explained by a serial pathway, whereby sex influences self/other-scrutiny, which reduces self-efficacy, and ultimately leads to heightened MPA. This study therefore tested the serial mediation model of sex → self/other-scrutiny → self-efficacy → MPA.

This study focuses on community choirs, an understudied context in MPA research. While most existing MPA studies have focused on conservatoire students and professional soloists, community choirs represent a distinct performance environment characterized by collective coordination, shared responsibility, and ongoing peer evaluation rather than formal assessment. Thus, this study aims to explore whether the mechanisms of MPA observed in other contexts generalize to this non-professional, socially embedded setting. By testing scrutiny-based mechanisms in Chinese community choirs, the study provides valuable cross-cultural evidence that extends our understanding of MPA beyond Western settings.

**Figure 1:**
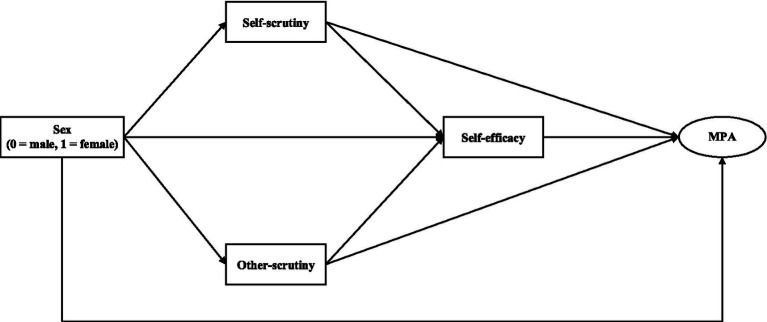
The hypothetical model. MPA, music performance anxiety.

Given the background, the present study aimed to investigate the associations between sex, self/other-scrutiny, self-efficacy, and MPA in a sample of Chinese choir members. We hypothesized that (H1) male and female would differ in the level of MPA, with female exhibiting higher MPA than males; (H2) self-scrutiny would mediate the association between sex and MPA; (H3) other-scrutiny would mediate the association between sex and MPA; (H4) self-efficacy would mediate the association between sex and MPA; (H5) self-scrutiny and self-efficacy would serially mediate the association between sex and MPA; (H6) other-scrutiny and self-efficacy would serially mediate the association between sex and MPA. The hypothetical model was shown in Figure 1.

## 2 Methods

### 2.1 Participants and data collection

This study adopted a cross-sectional survey design. Participants were recruited from several community-based choirs located in the central and western provinces of China. Data were collected between July and August 2025 using Wenjuanxing,^1^ a widely used and reliable online survey platform in China. Eligible respondents were active choir members aged 18 years or older who had participated in at least one public performance in the preceding year. The study focused on community-based choir members rather than professional musicians or advanced music students, in order to examine MPA processes in a non-professional, everyday musical performance context. Community choirs typically vary in size, rehearsal routines, conductor style, and performance frequency; however, the present study was designed to examine individual-level psychological processes across a broad community sample rather than to model choir-level differences. Individuals who self-reported severe psychiatric or neurological conditions, had significant sensory impairments affecting choir participation, or provided invalid responses were excluded. Participation was voluntary and anonymous, and electronic informed consent was obtained after participants had read the online information sheet. The study protocol was approved by the Research Ethics Committee of Xihua University (Approval No. XH250715-01).

A total of 804 individuals completed the survey. After excluding 30 cases that failed quality checks, the final analytic sample comprised 774 valid responses for statistical analyses.

### 2.2 Measures

#### 2.2.1 Demographic variables

Demographic information was collected to provide background characteristics of the sample. Variables included participants’ age, sex, educational level, self-reported family financial situation, and employment status.

#### 2.2.2 MPA

MPA was assessed using the Chinese version of the Stage Music Performance Anxiety Inventory (Su et al., 2017). This instrument evaluates anxiety specifically arising in stage performance situations and comprises two subscales: physiological anxiety (e.g., “I feel nervous”) and performance-related concerns (e.g., “I worry that today’s performance may go wrong”). The scale contains 14 items, with each rating on a 7-point Likert scale from 1 (never) to 7 (always). Higher scores reflect more severe symptoms of performance anxiety. In the current sample, the internal consistency of the overall scale was satisfactory, with a Cronbach’s alpha of 0.95. To complement alpha, McDonald’s omega was also calculated, yielding an *ω* value of 0.96, indicating excellent internal consistency.

#### 2.2.3 Self-scrutiny

Self-scrutiny was measured using 2 items adapted from the Revised Kenny Music Performance Anxiety Inventory (Kenny, 2009). The two items were “I worry about my own evaluation of the performance” and “I worry whether I performed well enough.” Responses were made on a 7-point Likert scale (1 = never to 7 = always), with higher scores reflecting greater concern about self-evaluation. These items were selected to capture a narrowly defined, situation-specific evaluative focus, consistent with prior uses of adapted K-MPAI items to assess specific cognitive components of MPA (Kenny, 2023). In the present study, the scale demonstrated excellent internal consistency, with a Cronbach’s alpha of 0.90. To complement alpha, McDonald’s omega was also computed, yielding an ω value of 0.91, indicating excellent reliability.

#### 2.2.4 Other-scrutiny

Other-scrutiny was assessed with a single item adapted from the Revised Kenny Music Performance Anxiety Inventory (Kenny, 2009): “I worry about being scrutinized by others.” Given the absence of a widely validated standalone scale specifically assessing other-scrutiny in musical performance contexts, a single-item measure was used to capture this concrete and experiential evaluative concern. Such an approach is consistent with methodological work suggesting that single-item measures can be informative when constructs are unidimensional and context-specific (Allen et al., 2022).

#### 2.2.5 Self-efficacy

Self-efficacy was assessed using the Chinese version of the General Self-Efficacy Scale (Schwarzer and Jerusalem, 2012). This instrument measures individuals’ perceived confidence in handling challenges and achieving goals in daily life. It consists of 10 items (e.g., “I can always manage to solve difficult problems if I try hard enough”), each rated on a 4-point Likert scale ranging from 1 (not at all true) to 4 (exactly true). Higher scores indicate stronger self-efficacy beliefs. The scale has been widely validated in Chinese samples and demonstrates good psychometric properties (Su et al., 2023). In the present study, the Cronbach’s alpha coefficient was 0.85. To complement alpha, McDonald’s omega was also calculated as an alternative reliability estimate, yielding an *ω* value of 0.87, indicating good internal consistency.

### 2.3 Statistical analysis

Descriptive analyses were first conducted to summarize sample characteristics. Pearson correlation coefficients were then calculated to examine the bivariate associations among self-scrutiny, other-scrutiny, self-efficacy, and MPA. Independent-samples *t*-tests were employed to assess sex differences in these key variables. To evaluate the hypothesized mediation pathways, structural equation modelling (SEM) was performed using the Maximum Likelihood estimator while controlling for background covariates. Standardized path coefficients (*β*) were reported for model estimation. Model adequacy was evaluated using conventional fit indices, with acceptable thresholds defined as Comparative Fit Index (CFI) ≥ 0.90, Tucker-Lewis Index (TLI) ≥ 0.90, Root Mean Square Error of Approximation (RMSEA) ≤ 0.08, and Standardized Root Mean Square Residual (SRMR) ≤ 0.08 (Kline, 2016). SEM were conducted with Mplus 8.3, whereas descriptive statistics, correlations, and *t*-tests were performed using SPSS 25.0. Statistical significance was set at *p* < 0.05 (two-tailed).

## 3 Results

### 3.1 Descriptive statistics

As shown in Table 1, among all participants, the mean age was 46.02 years (SD = 18.19). Approximately one-third were male (27.1%), and more than 70% held a college degree (72.0%). Regarding self-reported family economic status, 21.6% reported it as poor, whereas 12.3% rated it as excellent. In terms of employment, 22.7% identified as students and 28.2% were full-time employees.

**Table 1:** Participants characteristics.

Variable	n/mean	%/SD
Age	46.02	18.19
Sex		
Male	210	27.1
Female	564	72.9
Educational level		
High school or below	124	16.1
College	560	72.0
Master or above	90	11.6
Self-reported family financial situation		
Poor	167	21.6
Fair	122	15.8
Good	95	12.3
Very Good	54	7.0
Excellent	95	12.3
Not reported	241	31.1
Occupational status		
Students	176	22.7
Full-time employed	218	28.2
Not Employed	36	4.7
Not reported	344	44.4

### 3.2 Pearson correlations

As presented in Table 2, self-efficacy was significantly and negatively correlated with self-scrutiny, other-scrutiny, and MPA (*r* ranges from −0.11 to −0.07). In contrast, self-scrutiny, other-scrutiny, and MPA were significantly and positively correlated with each other (*r* ranges from 0.63 to 0.82).

**Table 2:** Pearson correlations.

Variable	1	2	3	4
Self-efficacy	1			
Self-scrutiny	−0.07^*^	1		
Other-scrutiny	−0.10^**^	0.82^**^	1	
MPA	−0.11^**^	0.65^**^	0.63^**^	1

*p < 0.05, **p < 0.01, ***p < 0.001. MPA, music performance anxiety.

### 3.3 Sex differences in the key studied variables

Males showed significantly higher mean scores (SD) than females in self-efficacy [36.47 (7.44) vs. 34.38 (5.71), *p* < 0.001, Cohen’s *d* = 0.32]. In contrast, females showed significantly higher mean scores (SD) than males in terms of self-scrutiny [5.72 (2.75) vs. 6.83 (3.05), *p* < 0.001, Cohen’s *d* = −0.38], other-scrutiny [3.12 (1.47) vs. 3.78 (1.74), *p* < 0.001, Cohen’s *d* = −0.41], and MPA [40.84 (16.18) vs. 45.08 (16.90), *p* = 0.001, Cohen’s *d* = −0.26]. Such information was presented in Table 3.

**Table 3:** Levels of the key studied variables by sex.

Variable	Male		Female		*p*	Cohen’s *d*
	Mean	SD	Mean	SD		
Self-efficacy	36.47	7.44	34.38	5.71	<0.001	0.32
Self-scrutiny	5.72	2.75	6.83	3.05	<0.001	−0.38
Other-scrutiny	3.12	1.47	3.78	1.74	<0.001	−0.41
MPA	40.84	16.18	45.08	16.90	0.001	−0.26

MPA, music performance anxiety.

### 3.4 SEM

**Figure 2:**
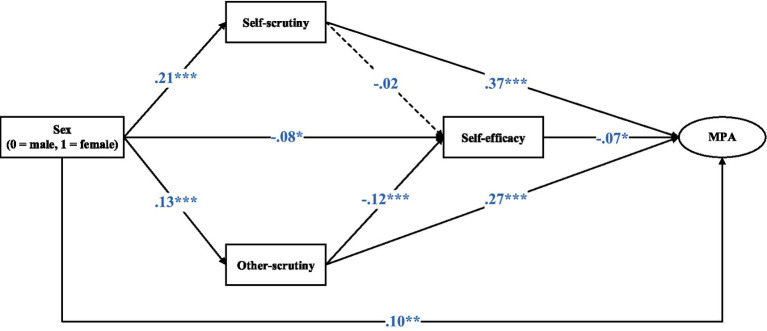
Structural equation modelling of the mediation effect of self-scrutiny, other-scrutiny, and self-efficacy between sex and MPA. MPA, music performance anxiety. *p < 0.05, **p < 0.01, ***p < 0.001.

Figure 2 and Table 4 present the results of the path analysis, which showed satisfactory model fit indices (CFI = 0.947, TLI = 0.927, SRMR = 0.035, RMSEA = 0.063). The five of six hypotheses were supported. (1) The direct effect between sex and MPA was statistically significant (*β* = 0.095; *p* = 0.003). (2) The indirect effect between sex and MPA via self-scrutiny was statistically significant (*β* = 0.078; 95% CI: 0.043–0.114; *p* < 0.001; effect size = 36.3%), i.e., sex was positively associated with self-scrutiny (*β* = 0.21, *p* < 0.001), which was in turn positively associated with MPA (*β* = 0.37, *p* < 0.001). (3) The indirect effect between sex and MPA via other-scrutiny was statistically significant (*β* = 0.035; 95% CI: 0.012–0.058; *p* < 0.001; effect size = 16.3%), i.e., sex was positively associated with other-scrutiny (*β* = 0.13, *p* < 0.001), which was in turn positively associated with MPA (*β* = 0.27, *p* < 0.001). (4) The indirect effect between sex and MPA via self-efficacy was statistically significant (*β* = 0.006; 95% CI: 0.002–0.010; *p* = 0.045; effect size = 2.8%), i.e., sex was negatively associated with self-efficacy (*β* = −0.08, *p* = 0.030), which was in turn negatively associated with MPA (*β* = −0.07, *p* = 0.010). (5) The serial mediation sex → other-scrutiny → self-efficacy → MPA was also statistically significant, but has a very small mediation effect size of 1.0% (*β* = 0.001; 95% CI: 0.0007–0.0013; *p* = 0.027). However, the serial mediation sex → self-scrutiny → self-efficacy → MPA was not statistically significant. Given that the direct effect of sex on MPA was statistically significant, the mediation effects only partially accounted for the observed sex differences.

**Table 4:** Mediation analysis.

Path	*β*	SE	*p*	95% CI	Mediation effect size
Direct path					
Sex → MPA	0.095	0.029	0.003	(0.065, 0.125)	–
Indirect path					
Sex → SS → MPA	0.078	0.024	<0.001	(0.043, 0.114)	36.3%
Sex → OS → MPA	0.035	0.019	<0.001	(0.012, 0.058)	16.3%
Sex → SE → MPA	0.006	0.004	0.045	(0.002, 0.010)	2.8%
Sex → SS → SE → MPA	0.0003	0.001	0.742	(−0.0006, 0.0018)	–
Sex → OS → SE → MPA	0.001	0.001	0.027	(0.0007, 0.0013)	1.0%

MPA, music performance anxiety; SS, self-scrutiny; OS, other-scrutiny; SE, self-efficacy.

## 4 Discussion

The present study examined sex differences in MPA among Chinese choir members, and investigated the mediating roles of self-scrutiny, other-scrutiny, and self-efficacy. Several important findings emerged. First, females reported significantly higher levels of MPA than males. Second, mediation analyses revealed that self-scrutiny accounted for the largest proportion (36%) of the indirect pathway between sex and MPA. Other-scrutiny also a mediator, but its effect size was smaller (16.3%) of the variance. These findings underscore the significance of both internal and external evaluative processes in the experience of MPA. Self-efficacy mediated only 2.8% of the sex effect, and the serial mediation pathways involving other-scrutiny explained merely 1% of the total effect size. These findings indicate that evaluative cognitive styles, particularly self-focused evaluative scrutiny, are central mechanisms underlying sex disparities in MPA. The results extend prior research by disentangling self- and other-directed evaluative concerns, while also testing self-efficacy as a resource factor, thereby advancing understanding of how gendered socialization contributes to musicians’ performance anxiety. Collectively, these results extend scrutiny-based models of MPA to an adult community choir context. Additionally, they provide cross-cultural evidence from China, contributing to the understanding of social-evaluative processes, self-regulation, and well-being in everyday music participation.

H1 was supported: females reported significantly higher levels of MPA than males, which aligns with extensive evidence (Papageorgi, 2020; Butković et al., 2021; Sokoli et al., 2022; Kayrouz et al., 2025). Several explanations have been proposed. From a gender role theory perspective, females are often socialized to be more emotionally expressive, interpersonally sensitive, and attuned to social evaluation, while males are encouraged to adopt agentic, task-focused roles (Eagly and Wood, 2016). This socialization pattern increases females’ vulnerability to performance anxiety in evaluative contexts such as choir performance. However, this pattern is not universal: some studies, particularly in younger samples, have reported no significant sex differences in MPA (Dempsey and Comeau, 2019), suggesting that the emergence and magnitude of sex differences may depend on developmental stage and the evaluative demands of the performance context.

H2 was supported, indicating that self-scrutiny significantly mediated the relationship between sex and MPA. Females reported higher levels of self-scrutiny, which was in turn associated with greater MPA, with this pathway accounting for more than one-third of the observed sex difference. This finding is consistent with gender role theory (Eagly and Wood, 2016), which suggests that gendered socialization fosters greater internalization of evaluative standards and self-monitoring among females. It also resonates with cognitive interference models, which posit that excessive self-focused evaluation disrupts attentional control during performance (Mellings and Alden, 2000; Johnson and Whisman, 2013). Females’ heightened self-scrutiny is consistent with prior work showing that females engage more frequently in rumination and self-critical thinking when under stress, which amplifies their vulnerability to anxiety (Lilly et al., 2023). Such self-scrutiny may involve heightened self-monitoring, exaggerated concern over mistakes, and doubts about adequacy, thereby intensifying MPA (Osborne et al., 2014; Nielsen et al., 2017). Importantly, this study is among the first to demonstrate quantitatively that self-scrutiny explains such a large proportion of the sex difference in MPA.

H3 was also supported, showing that other-scrutiny functioned as a significant mediator between sex and MPA. Females reported higher levels of concern about others’ evaluations, and this interpersonal vigilance was associated with increased performance anxiety. Although the magnitude of this indirect effect (16.3%) was less than half of that observed for self-scrutiny, the pathway remains noteworthy. This pattern aligns with research on fear of negative evaluation, which has been shown to be higher in females (Jia and Yue, 2023; Wei et al., 2025). While this effect is smaller, it still suggests that external evaluative concerns play a role in the overall experience of MPA. Consistent with gender role theory (Chaplin, 2015), the present findings suggest that sensitivity to others’ evaluation represents an interpersonal channel through which gendered expectations are expressed in performance contexts characterized by high social visibility. In choir performance, where individual contributions are embedded within a collective outcome, performers are especially exposed to social judgement and mutual accountability. Within a collectivist cultural setting, social evaluation carries broader interpersonal and normative significance, which may further amplify the anxiety-provoking effects of external scrutiny (Zhou and Zhang, 2024). These findings indicate that other-scrutiny contributes to sex differences in MPA through a context-dependent interpersonal mechanism that is theoretically distinct from the internalized evaluative processes captured by self-scrutiny.

H4 was supported, as self-efficacy significantly mediated the relationship between sex and MPA. Females reported lower levels of self-efficacy, and these diminished beliefs in their own capabilities were associated with higher performance anxiety. This finding is consistent with prior research highlighting self-efficacy as a protective factor that fosters adaptive coping and resilience in evaluative contexts (Ou and Qin, 2025). Nevertheless, in the present study, the mediating effect of self-efficacy was relatively small, accounting for only 2.8% of the sex difference in MPA. This modest effect size is theoretically plausible, given that self-efficacy reflects a relatively stable, generalized belief system that may be less sensitive to situational performance demands than immediate evaluative cognitions such as self- and other-scrutiny (Stojanovic et al., 2021). Accordingly, while self-efficacy contributes to individual differences in MPA, it appears to play a secondary role relative to more proximal cognitive processes operating in the performance context. This interpretation accords with previous evidence suggesting that although self-efficacy buffers against MPA, its influence is often overshadowed by more immediate cognitive and emotional responses to evaluative settings (Endler et al., 2001).

H5, which proposed that self-scrutiny and self-efficacy would serially mediate the association between sex and MPA, was not supported. Although females reported higher levels of self-scrutiny, the pathway from self-scrutiny to self-efficacy did not reach significance, preventing the emergence of a sequential mediation effect. This suggests that heightened self-critical attention, while detrimental to performance confidence in the moment, does not necessarily translate into a stable erosion of self-efficacy beliefs. From a theoretical standpoint, this pattern is consistent with cognitive interference models, which conceptualize self-focused evaluative attention as a situational process that disrupts momentary performance rather than reshaping enduring self-beliefs (Johnson and Whisman, 2013). One possible explanation is that self-efficacy reflects a broader and more enduring appraisal of one’s capabilities, shaped by accumulated mastery experiences, whereas self-scrutiny reflects an acute evaluative focus that operates at the situational level (Pfitzner-Eden, 2016). Consequently, self-scrutiny may exacerbate anxiety directly but may not reliably undermine generalized efficacy beliefs.

By contrast, H6 was supported: other-scrutiny and self-efficacy sequentially mediated the relationship between sex and MPA, although the effect size was modest (1.0%). Females’ greater sensitivity to external evaluation was associated with reduced self-efficacy, which in turn contributed to higher performance anxiety. This pathway highlights how concerns about others’ judgments may gradually erode broader confidence in one’s abilities, thereby amplifying vulnerability to MPA. Consistent with gender role theory (Shao et al., 2026), this pathway suggests that interpersonal evaluative concerns may represent a channel through which gendered expectations exert more enduring effects on confidence-related beliefs, rather than only triggering transient MPA. It should be noted that effects involving more distal psychological processes are often smaller in magnitude, as such associations are likely to reflect the combined influence of multiple intervening processes, contextual factors, and individual variability rather than a direct or unitary pathway (Shrout and Bolger, 2002). In the Chinese choral context, where performance is often collective and public, the salience of external evaluation may be especially pronounced. Repeated exposure to evaluative scrutiny could accumulate over time to diminish self-efficacy (Ou and Qin, 2025). These results indicate that self-scrutiny and other-scrutiny differ not only in focus (internal vs. external) but also in their temporal and theoretical functions: self-scrutiny operates primarily as an immediate cognitive interference mechanism, whereas other-scrutiny may exert more distal effects by shaping performers’ efficacy beliefs.

The present findings have several implications. Theoretically, the present study makes an important contribution to the MPA literature by quantifying and distinguishing the roles of self-scrutiny, other-scrutiny, and self-efficacy in explaining sex differences. While prior research has often relied on simple mean comparisons, our study advances scrutiny-based models of MPA by disentangling these mediators, providing a more nuanced understanding of how gendered socialization impacts MPA. These results highlight the critical role of internal evaluative processes, particularly self-scrutiny, in shaping MPA, thus contributing to the development of more sophisticated theoretical frameworks for MPA.

Practically, these findings offer valuable insights for both individual and group-level interventions. At the individual level, interventions should focus on reducing self-scrutiny by using cognitive-behavioral training to challenge perfectionism, mindfulness techniques to reduce rumination, and rehearsal practices that normalize mistakes (Shaw et al., 2020), as self-scrutiny explained approximately 36% of the sex difference in MPA. At the group level, structured exposure to performance settings and peer-based feedback can help mitigate the impact of external evaluation, as other-scrutiny accounted for 16.3% of the variance. Educators can foster supportive environments by promoting collective self-regulation and constructive peer feedback (Candia et al., 2023; Liu and Aryadoust, 2024). While self-efficacy explained only 2.8% of the sex difference, stepwise performance opportunities and supportive instruction can still enhance self-confidence and resilience to MPA over time (Zhang and Jenatabadi, 2024). In the context of Chinese choirs, cultural dynamics such as collective identity and “face” should be considered, with group-based approaches promoting social harmony and peer support. Given the modest effect sizes in serial mediation pathways, these interventions should be viewed as part of a broader, multi-faceted strategy for reducing MPA.

Despite its contributions, several limitations should be acknowledged. First, the cross-sectional design precludes conclusions about causal relationships. Longitudinal or experimental approaches are needed to clarify temporal ordering. Second, the reliance on self-report questionnaires may introduce reporting bias, and future studies could incorporate behavioral or physiological indicators. Third, self- and other-scrutiny were assessed using abbreviated measures, which may limit construct breadth and sensitivity. Future research should develop and validate more comprehensive multi-item scrutiny measures tailored to musical performance contexts. Fourth, the sample comprised Chinese adult community choir members. Although this context is highly relevant for community-based music participation, it may differ from other musical settings (e.g., solo performance, instrumentalists, professional ensembles, or formal conservatoire assessment) and from other cultural regions. Accordingly, the findings should be generalized primarily to similar amateur choral contexts, and future work should replicate the model across musical genres, performance formats, age groups, and cultures. Finally, there may be other mediating mechanisms underlying the association between sex and MPA that were not examined here, and future research should investigate these additional pathways.

## 5 Conclusion

In sum, this study demonstrates that females report higher levels of MPA than males. Self-scrutiny, other-scrutiny, and self-efficacy partially explain this disparity, with markedly different effect size. Self-scrutiny emerged as the dominant mediator, highlighting the critical role of internalized evaluative cognitions, while other-scrutiny also contributed but to a lesser extent. Self-efficacy accounted for a minimal portion of the sex difference. While these mechanisms were statistically significant, their practical relevance differs markedly, and conclusions should be weighted primarily toward pathways with larger and more proximal effects. These findings underscore the importance of addressing evaluative scrutiny in MPA and in practical interventions aimed at reducing anxiety among musicians. By situating the results within broader theories of gender role socialization and cognitive interference, and by considering the cultural context of Chinese choirs, the study contributes to a deeper understanding of the mechanisms underlying sex differences in MPA.

## Data Availability

The raw data supporting the conclusions of this article will be made available by the authors, without undue reservation.

## Footnotes

1
www.wjx.cn

## References

[ref1] Allen M. S. Iliescu D. Greiff S. (2022). Single item measures in psychological science: a call to action. Eur. J. Psychol. Assess. 38, 1–5. doi: 10.1027/1015-5759/a000699

[ref2] Barros S. Marinho H. França A. Pereira A. (2024). Music performance anxiety: a study of anxiety predictors in higher education music students in Portugal. Int. J. Music. Educ. doi: 10.1177/02557614241280040

[ref3] Butković A. Vukojević N. Carević S. (2021). Music performance anxiety and perfectionism in Croatian musicians. Psychol. Music 50, 100–110. doi: 10.1177/0305735620978692

[ref4] Candia V. Kusserow M. Margulies O. Hildebrandt H. (2023). Repeated stage exposure reduces music performance anxiety. Front. Psychol. 14:1146405. doi: 10.3389/fpsyg.2023.1146405, 37020906 PMC10067860

[ref5] Chaplin T. M. (2015). Gender and emotion expression: a developmental contextual perspective. Emot. Rev. 7, 14–21. doi: 10.1177/1754073914544408, 26089983 PMC4469291

[ref6] Cornett V. Urhan G. (2021). Performance anxiety experiences and coping techniques of Turkish music students and their teachers. Int. J. Music. Educ. 39, 504–519. doi: 10.1177/02557614211005907

[ref7] Dempsey E. Comeau G. (2019). Music performance anxiety and self-efficacy in young musicians: effects of gender and age. Music Perform. Res. 9, 60–79.

[ref8] Dobos B. Piko B. F. Kenny D. T. (2019). Music performance anxiety and its relationship with social phobia and dimensions of perfectionism. Res. Stud. Music Educ. 41, 310–326. doi: 10.1177/1321103X18804295

[ref9] Du H. Liu Y. Sun J. (2025). Psychological resilience and music performance anxiety: exploring mediators and sex differences in Chinese choir members. Front. Psychol. 16:1703571. doi: 10.3389/fpsyg.2025.1703571, 41194898 PMC12584046

[ref10] Eagly A. H. Wood W. (2016). Social role theory of sex differences. Naples, N.A: The Wiley Blackwell Encyclopedia of Gender and Sexuality Studies, 1–3.

[ref11] Endler N. S. Speer R. L. Johnson J. M. Flett G. L. (2001). General self-efficacy and control in relation to anxiety and cognitive performance. Curr. Psychol. 20, 36–52. doi: 10.1007/s12144-001-1002-7

[ref12] Eysenck M. W. Derakshan N. Santos R. Calvo M. G. (2007). Anxiety and cognitive performance: attentional control theory. Emotion 7, 336–353. doi: 10.1037/1528-3542.7.2.336 17516812

[ref13] Guyon A. J. A. A. Hildebrandt H. Güsewell A. Horsch A. Nater U. M. Gomez P. (2022). How audience and general music performance anxiety affect classical music students’ flow experience: a close look at its dimensions. Front. Psychol. 13:959190. doi: 10.3389/fpsyg.2022.959190, 36389478 PMC9649719

[ref14] Herman R. Clark T. (2023). It’s not a virus! Reconceptualizing and de-pathologizing music performance anxiety. Front. Psychol. 14:1194873. doi: 10.3389/fpsyg.2023.1194873, 38022988 PMC10667921

[ref15] Jia Y. Yue Y. (2023). Fear of positive evaluation mediates the relationship between self-efficacy and fear of negative evaluation in nursing students: a cross-sectional study. J. Prof. Nurs. 47, 88–94. doi: 10.1016/j.profnurs.2023.04.007 37295917

[ref16] Johnson D. P. Whisman M. A. (2013). Gender differences in rumination: a meta-analysis. Pers. Individ. Dif. 55, 367–374. doi: 10.1016/j.paid.2013.03.019, 24089583 PMC3786159

[ref17] Kayrouz R. Karin E. Staples L. Dear B. Nielssen O. Titov N. (2025). A review of the 257 meta-analyses of the differences between females and males in prevalence and risk, protective factors, and treatment outcomes for mental disorder. BMC Psychiatry 25:677. doi: 10.1186/s12888-025-06848-7, 40610965 PMC12224573

[ref18] Kenny D. T. (2009). “The factor structure of the revised Kenny music performance anxiety inventory” in International symposium on performance science ed. Williamon A. (Utrecht: Association Européenne des Conservatoires), 37–41.

[ref19] Kenny D. T. (2023). The Kenny music performance anxiety inventory (K-MPAI): scale construction, cross-cultural validation, theoretical underpinnings, and diagnostic and therapeutic utility. Front. Psychol. 14:1143359. doi: 10.3389/fpsyg.2023.1143359, 37325731 PMC10262052

[ref20] Kinney C. Saville P. Heiderscheit A. Himmerich H. (2025). Therapeutic interventions for music performance anxiety: a systematic review and narrative synthesis. Behav. Sci. 15:138. doi: 10.3390/bs15020138, 40001769 PMC11851691

[ref21] Kline R. B. (2016). Principles and practice of structural equation modeling. 4th Edn. New York, NY, US: Guilford Press.

[ref22] Kring A. M. Gordon A. H. (1998). Sex differences in emotion: expression, experience, and physiology. J. Pers. Soc. Psychol. 74, 686–703, 9523412 10.1037//0022-3514.74.3.686

[ref23] Lilly K. J. Howard C. Zubielevitch E. Sibley C. G. (2023). Thinking twice: examining gender differences in repetitive negative thinking across the adult lifespan. Front. Psychol. 14:1239112. doi: 10.3389/fpsyg.2023.1239112, 38022916 PMC10663279

[ref24] Liu T. Aryadoust V. (2024). Orchestrating teacher, peer, and self-feedback to enhance learners’ cognitive, behavioral, and emotional engagement and public speaking competence. Behav. Sci. (Basel) 14:725. doi: 10.3390/bs14080725, 39199121 PMC11351891

[ref25] Lu H. Yang J. Zhao K. Jin Z. Wen X. Hu N. . (2024a). Perceived risk of COVID-19 hurts mental health: the mediating role of fear of COVID-19 and the moderating role of resilience. BMC Psychiatry 24:58. doi: 10.1186/s12888-024-05511-x 38254008 PMC10802027

[ref26] Lu H. Yu Y. Wang D. B. Wu A. M. S. Chen J. H. Zhang G. . (2024b). Association between interpersonal resources and mental health professional help-seeking among Chinese adolescents with probable depression: mediations via personal resources and active coping. BMC Psychiatry 24:840. doi: 10.1186/s12888-024-06271-4, 39574049 PMC11580335

[ref27] Malespina A. Seifollahi F. Singh C. (2024). Bioscience students in physics courses with higher test anxiety have lower grades on high-stakes assessments, and women report more test anxiety than men. Educ. Sci. 14:1092. doi: 10.3390/educsci14101092

[ref28] Mellings T. M. Alden L. E. (2000). Cognitive processes in social anxiety: the effects of self-focus, rumination and anticipatory processing. Behav. Res. Ther. 38, 243–257. doi: 10.1016/s0005-7967(99)00040-6, 10665158

[ref29] Moraga-Pumarino A. Salvo-Garrido S. Ortiz-Cea V. (2025). Gender, self-efficacy, and academic performance: evidence in business education program. Behav. Sci. 15:563. doi: 10.3390/bs15050563, 40426341 PMC12109101

[ref30] Nicholl T. J. Abbott M. J. (2024). Debilitating performance anxiety in musicians and the performance specifier for social anxiety disorder: should we be playing the same tune? Clin. Psychol. 28, 331–345. doi: 10.1080/13284207.2024.2402339

[ref31] Nielsen C. Studer R. K. Hildebrandt H. Nater U. M. Wild P. Danuser B. . (2017). The relationship between music performance anxiety, subjective performance quality and post-event rumination among music students. Psychol. Music 46, 136–152. doi: 10.1177/0305735617706539

[ref32] Osborne M. S. Greene D. J. Immel D. T. (2014). Managing performance anxiety and improving mental skills in conservatoire students through performance psychology training: a pilot study. Psychol. Well-Being 4:18. doi: 10.1186/s13612-014-0018-3

[ref33] Ou J. Qin C. (2025). Exploring musical self-efficacy and performance anxiety in young violin learners: insights from mainland China. Front. Psychol. 16:1575591. doi: 10.3389/fpsyg.2025.1575591, 40636046 PMC12239020

[ref34] Papageorgi I. (2020). Prevalence and predictors of music performance anxiety in adolescent learners: contributions of individual, task-related and environmental factors. Musicae Sci. 26, 101–122. doi: 10.1177/1029864920923128

[ref35] Pfitzner-Eden F. (2016). Why do I feel more confident? Bandura’s sources predict preservice teachers’ latent changes in teacher self-efficacy. Front. Psychol. 7:1486. doi: 10.3389/fpsyg.2016.01486, 27807422 PMC5070217

[ref36] Schwarzer R. Jerusalem M. (2012). General self-efficacy scale. APA PsycTests. doi: 10.1037/t00393-000

[ref37] Shao H. Du M. Wang D. B. Wu X. Ren Z. Yu Y. . (2026). Sex differences in social anxiety: a longitudinal serial mediation via perceived stress and resilience among adolescents in China. J. Affect. Disord. 392:120212. doi: 10.1016/j.jad.2025.120212, 40912330

[ref38] Shaw T. A. Juncos D. G. Winter D. (2020). Piloting a new model for treating music performance anxiety: training a singing teacher to use acceptance and commitment coaching with a student. Front. Psychol. 11:882. doi: 10.3389/fpsyg.2020.00882, 32547438 PMC7270208

[ref39] Shawon M. S. R. Hossain F. B. Hasan M. Rahman M. R. (2024). Gender differences in the prevalence of anxiety and depression and care seeking for mental health problems in Nepal: analysis of nationally representative survey data. Glob Ment Health (Camb) 11:e46. doi: 10.1017/gmh.2024.37, 38690568 PMC11058515

[ref40] Shrout P. Bolger N. (2002). Mediation in experimental and nonexperimental studies: new procedures and recommendations. Psychol. Methods 7, 422–445. doi: 10.1037/1082-989X.7.4.422, 12530702

[ref41] Sokoli E. Hildebrandt H. Gomez P. (2022). Classical music students’ pre-performance anxiety, catastrophizing, and bodily complaints vary by age, gender, and instrument and predict self-rated performance quality. Front. Psychol. 13:905680. doi: 10.3389/fpsyg.2022.905680, 35814093 PMC9263585

[ref42] Stojanovic M. Fries S. Grund A. (2021). Self-efficacy in habit building: how general and habit-specific self-efficacy influence behavioral automatization and motivational interference. Front. Psychol. 12:643753. doi: 10.3389/fpsyg.2021.643753, 34025512 PMC8137900

[ref43] Su Y.-H. Ho Y.-C. Cheng Y.-R. Chen H.-S. (2017). The development of “anticipatory music performance anxiety inventory” and “stage music performance anxiety inventory.”. Psychol. Test. 64, 207–235. doi: 10.7108/PT.201709_64(3).0002

[ref44] Su H. Zhou Y. Sun Y. Cai Y. (2023). The relationship between depression and subjective cognitive decline in older adults of China: the mediating role of general self-efficacy. Psychol. Health Med. 28, 1057–1067. doi: 10.1080/13548506.2022.2125165, 36165717

[ref45] Wei Y. Chen H. Sun B. Kong L. (2025). The relationship between physical appearance perfectionism on subthreshold depression in college students: the role of gender and fear of negative evaluation. Front. Public Health 13:1559815. doi: 10.3389/fpubh.2025.1559815, 40206157 PMC11978643

[ref46] Wine J. (1971). Test anxiety and direction of attention. Psychol. Bull. 76, 92–104. doi: 10.1037/h0031332, 4937878

[ref47] Yu Z. Deng X. (2022). A Meta-analysis of gender differences in e-learners’ self-efficacy, satisfaction, motivation, attitude, and performance across the world. Front. Psychol. 13:897327. doi: 10.3389/fpsyg.2022.897327, 35664150 PMC9159470

[ref48] Zhang H. Jenatabadi H. S. (2024). Effects of social support on music performance anxiety among university music students: chain mediation of emotional intelligence and self-efficacy. Front. Psychol. 15:1389681. doi: 10.3389/fpsyg.2024.1389681 39377059 PMC11457729

[ref49] Zhou B. Zhang S. (2024). Exploring Mianzi consciousness congruence and its impact on unethical pro-organizational behavior. BMC Psychol. 12:436. doi: 10.1186/s40359-024-01934-z, 39135140 PMC11320878

